# Runs of homozygosity analysis for selection signatures in the Yellow Korean native chicken

**DOI:** 10.5713/ab.24.0092

**Published:** 2024-05-07

**Authors:** Jaewon Kim, John Kariuki Macharia, Minjun Kim, Jung Min Heo, Myunghwan Yu, Hyo Jun Choo, Jun Heon Lee

**Affiliations:** 1 Division of Animal and Dairy Science, Chungnam National University, Daejeon 34134, Korea; 2 Poultry Research Institute, National Institute of Animal Science, Rural Development Administration, Pyeongchang 25342, Korea

**Keywords:** Local Chicken Breed, ROH Island, Runs of Homozygosity, Selection Signature Analysis, Yellow Korean Native Chicken

## Abstract

**Objective:**

Yellow Korean native chicken (KNC-Y) is one of the five pure Korean indigenous chicken breeds that were restored through a government project in 1992. KNC-Y is recognized for its superior egg production performance compared to other KNC lines. In this study, we performed runs of homozygosity (ROH) analysis to discover selection signatures associated with egg production traits in the KNC-Y population.

**Methods:**

A total of 675 DNA samples from KNC-Y were genotyped to generate single nucleotide polymorphism (SNP) data using custom 60K Affymetrix SNP chips. ROH analysis was performed using PLINK software, with predefined parameters set for the analysis. The threshold of ROH island was defined as the top 1% frequency of SNPs withing the ROH among the population.

**Results:**

In the KNC-Y population, a total of 29,958 runs of homozygosity (ROH) fragments were identified. The average total length of ROH was 120.84 Mb, with each ROH fragment having an average length of 2.71 Mb. The calculated ROH-based inbreeding coefficient (*F_ROH_*) was 0.13. Furthermore, we revealed the presence of ROH islands on chromosomes 1, 2, 4, 5, 7, 8, and 11. Within the identified regions, a total of 111 genes were annotated, and among them were genes related to economic traits, including *PRMT3*, *ANO5*, *HDAC4*, *LSS*, *PLA2G4A*, and *PTGS2*. Most of the overlapping quantitative trait locus regions with ROH islands were found to be associated with production traits.

**Conclusion:**

This study conducted a comprehensive analysis of ROH in the KNC-Y population. Notably, among the findings, the *PTGS2* gene is believed to play a crucial role in influencing the laying performance of KNC-Y.

## INTRODUCTION

Korean native chicken (KNC) breeds are indigenous breeds that have long been adapted to the Korean Peninsula. Unfortunately, the KNC population declined due to the Korean War and the introduction of foreign breeds, nearly disappearing by the 1970s. However, the increase in national income in the 1980s due to economic development resulted in a surge in domestic poultry consumption, promoting renewed public interest in KNCs. Consequently, in 1992, the National Institute of Animal Science (NIAS) launched a project to restore Korean traditional chicken breeds [1]. Over 15 years, the successful project identified five KNC lines based on different feather colors and body shapes: KNC-White (KNC-W), KNC-Black (KNC-B), KNC-Grey (KNC-G), KNC-Red (KNC-R), and KNC-Yellow (KNC-Y) [2]. These lines were officially registered in the Domestic Animal Diversity Information System (DAD-IS) of the Food and Agriculture Organization (FAO) [3]. Despite these achievements, KNCs continue to experience challenges related to their low egg production and slow growth compared with foreign commercial breeds. Therefore, enhancements of their economic traits are needed to reduce the dependence on imported foreign breeds. Such enhancements can be achieved through selective breeding strategies that consider the genetic characteristics associated with economic traits for each chicken line [4]. The KNC-Y has a plumage color similar to the KNC-R but is much lighter [1]. The results of population structure analyses using KNC SNP chip data have revealed that KNC-Y possesses unique genetic characteristics that distinguish it from other KNC breeds. Moreover, genetic distances have shown that KNC-Y is closely related to the KNC-W and KNC-R [5]. In a previous study, KNC-Y demonstrated better egg production traits compared with KNC lines [1]. Based on this, NIAS is conducting research to improve the performance of KNC-Y as a native layer chicken breed. Although a few studies have used cross-combination tests to enhance laying performance in KNCs, there has been minimal research focused on potential genetic characteristics [6,7].

Domesticated animals have undergone selection to im prove their economic traits. This selection has led to distinct characteristics in the genomic regions subjected to selection, including the fixation of allele frequency and occurrence of homozygous genotypes. These features are enhanced during continuous selection along with adjacent genes, creating extended haplotypes that exhibit reduced diversity. Additionally, the use of consanguineous mating during selection has resulted in long homozygous DNA segments in the genome. Continuous homozygous segments uninterrupted by heterozygous alleles are known as runs of homozygosity (ROH) [8,9]. ROH analysis enables the precise calculation of the inbreeding coefficient by identifying the proportion of ROH in the genome. Furthermore, the ROH distribution can serve as a marker to elucidate demographic phenomena in a population, such as genetic drift and population bottlenecks [8,10]. Overlapping ROH regions, commonly observed among individuals within a population, are known as ROH islands. These regions provide evidence of positive selection and can be used to identify potential genetic signatures [10]. In this study, a comprehensive ROH analysis was conducted to identify characteristic patterns of ROH in the genome and uncover selection signatures specific to KNC-Y, with a focus on egg production traits.

## MATERIALS AND METHODS

### Ethics statement

The animal study protocol was approved by Institution of Animal Care and Use Committee of the National Institute of Animal Science (Approval No.: NIAS2021-0525).

### Single nucleotide polymorphism genotyping and quality control of single nucleotide polymorphism chips

KNC-Y chickens from the Poultry Research Institute of NIAS were analyzed in this study. Blood samples were collected from 675 KNC-Y chickens and stored in ethylenediaminetetraacetic acid tubes. Genomic DNA was extracted using a genomic DNA extraction kit (GeNet Bio, Daejeon, Korea). DNA quality was checked using a NanoDrop2000c spectrophotometer (Thermos Scientific, Waltham, MA, USA) and samples were stored at −20°C until genotyping. Custom 60K Affymetrix SNP chips with 66,852 single nucleotide polymorphisms (SNPs) were used for SNP genotyping. SNPs were excluded based on the following criteria: Hardy–Weinberg test >1×10^−6^ (2,561 SNPs), non-autosomal SNPs (849 SNPs), call rate <0.9 (647 SNPs), and genotyping error rate <0.9 (one sample). Minor allele frequency filtering was not performed for the ROH analysis [11]. After application of the filtering criteria, 674 samples and 62,795 SNPs were included in subsequent analyses.

### Criteria establishment and runs of homozygosity analysis

**Figure 1:**
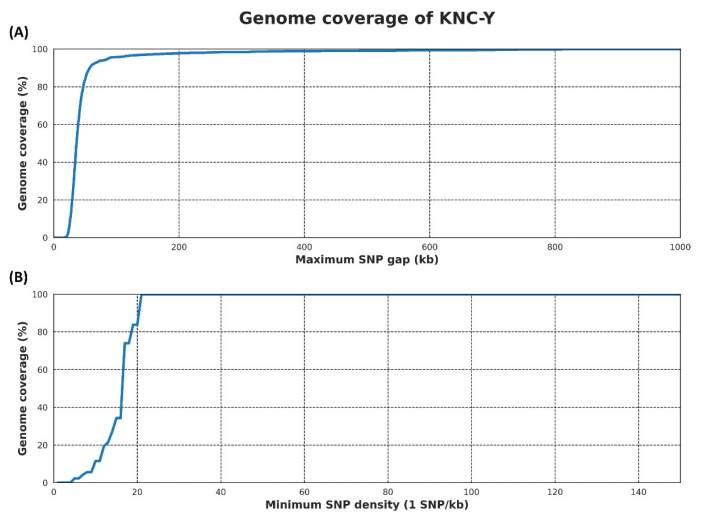
Genome coverage (%) results of ROH analysis in PLINK by manipulating maximum SNP gap (kb) and minimum SNP density (1 SNP/kb) parameters. (A) Genome coverage of maximum SNP gap reached 99.9% coverage at 820 and (B) genome coverage of minimum SNP density reached 99.9% coverage at 1 SNP/23 kb. ROH, runs of homozygosity; SNP, single nucleotide polymorphism.

The “--homozyg” command in PLINK v.1.9 was used to perform ROH analysis. Specific criteria were established based on published guidelines [10,11]. The minimum number of SNPs (--homozyg-snp) was calculated using the equation for l proposed by Lencz et al [12] and modified by Purfield et al [13]:


$$l=\frac{ln\frac{a}{n_{s}\times n_{i}}}{ln(1-\bar{het})}$$

where (het) $\bar{het}$ is the mean observed heterozygosity in the population calculated with the “--hardy” command, *n_s_* is the number of genotyped SNPs, *n_i_* is the number of animals studied, and *a* is the predetermined false positive rate for ROH (e.g., 0.05). Using this equation, *l* was calculated as approximately 57. The minimum number of SNPs in a window (--homozyg-window-snp) was set to the same value as the minimum number of SNPs [11]. One heterozygous SNP and one missing SNP were permitted per window. The minimum ROH length was set to 1 Mb, thus excluding ROH produced by linkage disequilibrium [14]. The maximum gap between two SNPs within an ROH (--homozyg-gap) and the minimum SNP density within an ROH (--homozyg-density) were determined using the genome coverage method [11]. Genome coverage represents the proportion of the total length of detectable ROH to the length of the completely homozygous genome. The genome coverage of a completely homozygous sample was calculated by varying the maximum gap from 1 to 1,000 kb and the minimum SNP density from 1 SNP/1 to 150 kb. After the genome coverage results had been reviewed, the maximum SNP gap was set to 820 kb, thus achieving 99.9% genome coverage (Figure 1A). The minimum SNP density was set to 1 SNP/50 kb, the default value of PLINK, because maximum genome coverage was observed at 1 SNP/23 kb, which is below the default value of PLINK (Figure 1B). ROH identified through analysis were categorized into five length classes (1 to 2, 2 to 4, 4 to 8, 8 to 16, and >16 Mb). The ROH-based inbreeding coefficient (FROH) was calculated using the following formula [15]:


$$\text{FROH}=\frac{L_{ROH}}{L_{Aut}}$$

where *L_ROH_* is the total ROH length and *L_Aut_* is the total genome length.

### Annotation of candidate genes and quantitative trait locus in runs of homozygosity islands

An ROH island was characterized as the genomic region exceeding the top 1% frequency of SNPs within the ROH among all individuals [9]. Candidate genes within an ROH island were annotated using BioMart in Ensembl, based on *Gallus gallus* SNP annotation information (GRCg6a.103) [16]. The GALLO package [17] in R software was used to identify quantitative trait locus (QTL) regions that overlapped with the ROH island region. QTL information was determined using the *G. gallus* genome annotation file (release 52, GRCg6a) from the QTL database [18].

## RESULTS AND DISCUSSION

### Establishment of criteria for runs of homozygosity analysis

Before an ROH analysis is conducted in PLINK, several parameters must be predefined. However, the default values provided by PLINK may not be universally suitable, depending on the species of interest and density of the SNP chip [11]. To address this issue, the maximum SNP gap value and minimum SNP density in ROH suitable for 60K SNP chip data were determined based on genome coverage. Genome coverage rapidly increased in the maximum SNP gap within the ROH, beginning at 30 kb and reaching a peak of 99.9% at 820 kb (Figure 1A). This value is below the default setting of 1,000 kb suggested by PLINK for ROH analysis. The selection of an unsuitable gap between SNPs can affect ROH analysis; thus, the gap must be set to the minimum value to ensure maximum genome coverage [11]. The default SNP density within the ROH in PLINK, set to 1 SNP/50 kb, is determined based on the average SNP density across several medium density SNP chips in various species. Therefore, it is possible that genomic regions with a density lower than this value will not be analyzed in an accurate manner [11]. Evaluation of the genome coverage of the minimum SNP density within the ROH of the 60K SNP chip used for KNC-Y confirmed that the maximum genome coverage was 1 SNP/23 kb (Figure 1B). Because this density exceeds the default value of 1 SNP/50 kb in PLINK, it was assumed not to adversely affect the analysis. Consequently, the minimum SNP density in ROH was maintained at 1 SNP/50 kb.

### Detection of runs of homozygosity in Yellow Korean native chicken

**Figure 2:**
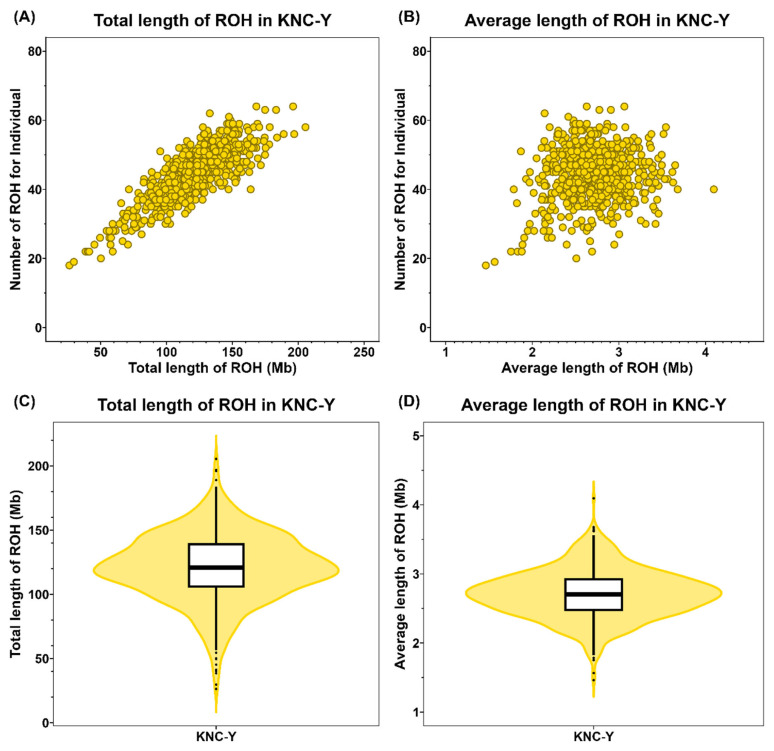
Length distribution of detected ROH in population. (A) The number of detected ROH and total length of ROH (Mb) per individual, (B) the number of detected ROH and average length of ROH in population (Mb) per individual, (C) the range of total ROH length in population, and (D) range of average length in population. ROH, runs of homozygosity.

**Figure 3:**
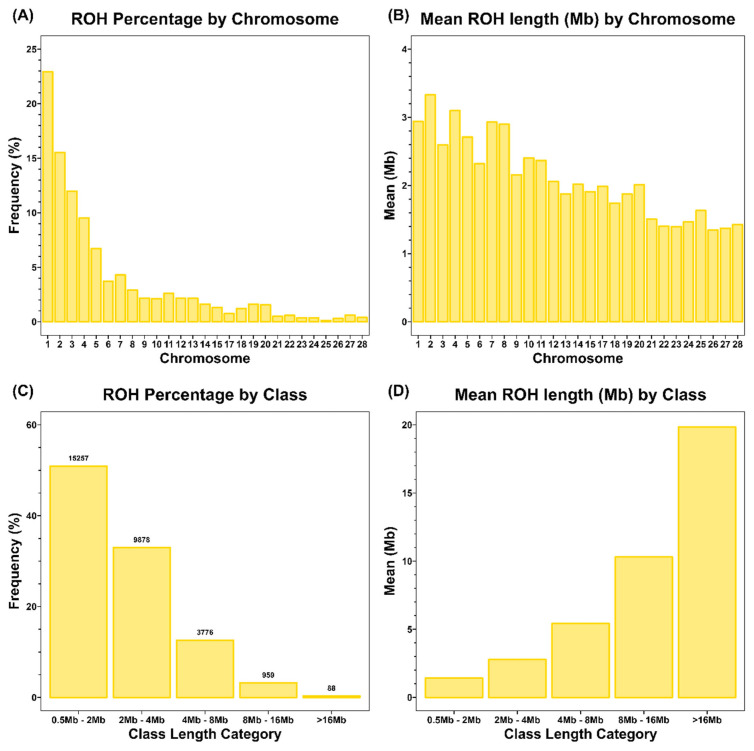
Distribution of ROH per chromosomes and proportion in different ROH length classes in Yellow Korean native chicken. (A) Percentage of ROH per chromosomes, (B) mean length of ROH per chromosomes, (C) proportion of total ROH in different length classes, and (D) mean length of ROH in different length classes. ROH, runs of homozygosity.

In KNC-Y, 29,958 ROH fragments were detected, with an average of 44 ROH per individual. The average total ROH length was approximately 120.84 Mb, the average length of each ROH fragment was 2.71 Mb, and *F_ROH_* was approximately 0.13 (Table 1). The total ROH length varied among individuals, ranging from 26.34 to 205.43 Mb; average ROH lengths ranged from 1.46 to 4.09 Mb (Figure 2). Notably, 23% of all ROH were identified on chromosome 1, revealing a tendency toward fewer ROH on other chromosomes (Figure 3A). The average ROH length for each chromosome was in the order of chromosome 2 (3.32 Mb)>chromosome 4 (3.09 Mb)>chromosome 1 (2.93 Mb) (Figure 3B). These findings indicate that although chromosome 1 had the highest ROH number, chromosomes 2 and 4 had longer ROH segments than chromosome 1. Examination of the five classes according to ROH length showed that ROH segments shorter than 4 Mb were predominant (83%) (Figure 3C). Data from low-density SNP chips, such as 60K chips, tend to overestimate the detection of ROH with sizes ≤4 Mb compared with high-density SNP chips. However, the ability to identify long ROH, specifically with sizes of ≥8 Mb, is similar to the ability observed with high-density chips [19]. Therefore, the number of true ROH with sizes <4 Mb is overestimated. ROH with sizes ≥8 Mb constituted 3.2% of all ROH. The average lengths for the ROH categories were 1.43, 2.78, 5.41, 10.29, and 19.84 Mb for the 1 to 2, 2 to 4, 4 to 8, 8 to 16, and >16 Mb categories, respectively; the longest ROH was 33.43 Mb on chromosome 1 (Figure 3D). ROH with sizes >8 Mb typically originate from a common ancestor within the past six generations, indicating recent inbreeding, whereas ROH with sizes ≤8 Mb are associated with a common ancestor more than six generations distant [20]. These findings indicate that some ROH in KNC-Y chickens were formed through recent inbreeding.

### Candidate genes and qunatitative trait loci in runs of homozygosity islands

**Figure 4:**
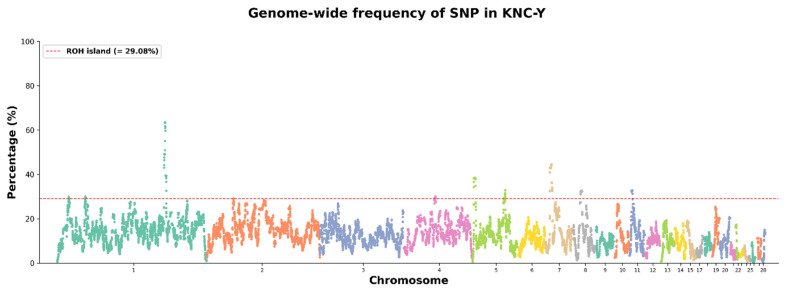
Manhattan plot of SNP frequencies in ROH across the genome. The red line represents the ROH island threshold for the top 1% of SNP frequency. SNP, single nucleotide polymorphism; ROH, runs of homozygosity.

**Figure 5:**
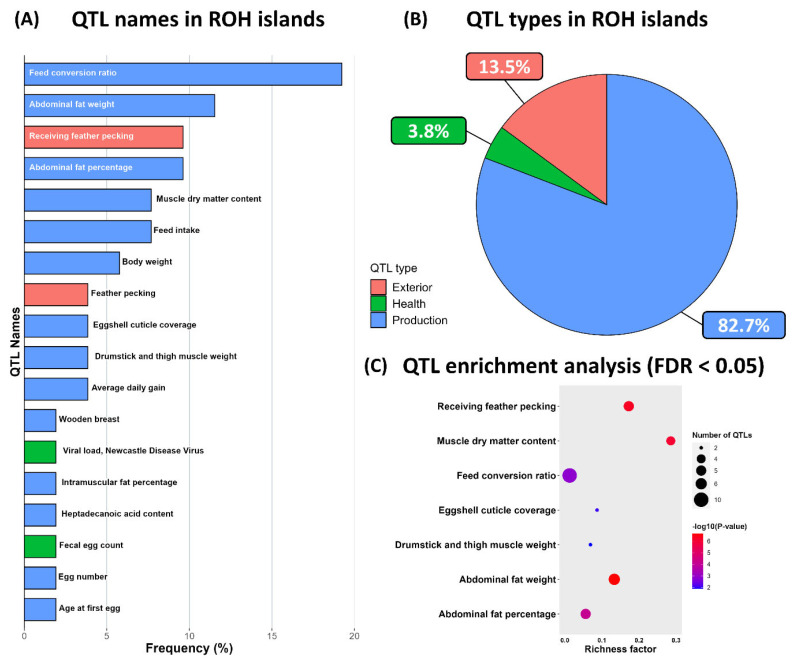
Identified QTLs in ROH islands based on the chicken Animal QTL database. (A) Distribution of related QTL names ratio in ROH islands, (B) proportion of related QTL types in ROH islands, and (C) enrichment analysis results for QTLs (false discovery rate <0.05) in ROH islands. QTL, quantitative trait loci; ROH, runs of homozygosity.

Arrangements of ROH in the analyzed genomes can reveal overlapping ROH regions. A homozygous region found in most individuals within a population is referred to as an ROH island [8]. An ROH island is a genomic region characterized by reduced genetic diversity due to selection; therefore, it is a valuable marker that can be used to identify regions associated with specific traits under selection [10]. The threshold for identifying ROH islands was regarding as a region with a frequency exceeding 29.08%, corresponding to the top 1% of SNPs among the 62,795 SNPs within the ROH (Figure 4). Thirteen ROH islands were distributed across seven chromosomes. The shortest ROH island had a length of 17 kb on chromosome 1, whereas the longest ROH island was 3,263 kb on chromosome 5. Chromosomes 1 and 5 had the most ROH islands (five each). In total, 111 genes were annotated in the ROH island regions (Table 2). The identification of ROH islands without annotated genes suggests that they arose from the selection of fixed noncoding DNA regions or regions involved in regulating gene expression [21]. The ROH island with the highest SNP frequency was on chromosome 1; it contained genes such as *TNSF13B*, *LIG4*, and *ARGLU1*. The *TNSF13B* and *LIG4* genes are associated with immune responses and the repair of damaged DNA; they play crucial roles in chicken survival [22,23]. Several candidate genes related to economic production traits were identified. *PRMT3* is involved in regulating transcription factors related to muscle growth [24]. *ANO5* encodes a membrane glycoprotein abundant in muscle cells and osteocytes; it is associated with muscle development [25]. *HDAC4*, encoding histone deacetylase, regulates gene transcription associated with muscle growth in chickens [26]. The *LSS* and *PLA2G4A* genes are involved in cholesterol and steroid biosynthesis; they have key roles in fat accumulation [27,28]. The *PTGS2* gene is involved in prostaglandin synthesis, which produces luteinizing hormone (LH) [28]. Consequently, the prostaglandin produced by the *PTGS2* gene is important for follicle maturation and development, influencing the LH surge [29,30]. To validate the characteristics of the ROH islands, QTL regions overlapping with the ROH islands were confirmed. When QTLs were categorized based on associated traits, 82.7% of QTLs were related to production traits, including the feed conversion rate, egg number, and age at first egg (Figure 5A, 5B). However, the QTLs associated with egg production trait ratios were lower compared with other production traits. This difference might be attributed to the relatively recent implementation of selection breeding strategies for layer lines. The results of QTL enrichment analysis revealed five QTLs with false discovery rate values of <0.05; the most notable QTL was related to muscle dry matter content. These findings suggest that the ROH islands in KNC-Y formed through selection aimed at improving productivity as domesticated breeds. Although the *PTGS2* gene might contribute to laying performance in KNC-Y, further research is needed to determine whether *PTGS2* is a unique characteristic of the KNC-Y breed alone by comparing the ROH island patterns with other KNC breeds.

## Figures and Tables

**Table 1:** Descriptive statistics for runs of homozygosity of Yellow Korean native chicken

No	H_O_±SD	Mean±SD			

		N_ROH_	S_ROH_ (Mb)	L_ROH_ (Mb)	F_ROH_
674	0.301±0.180	44±7.5	120.84±26.35	2.71±0.35	0.130±0.028

ROH, runs of homozygosity; No, number of individuals in population; H_O_, observed heterozygosity; SD, standard deviation; N_ROH_, number of ROH; S_ROH_, total length of ROH; L_ROH_, average length of ROH; F_ROH_, inbreeding coefficient based on ROH.

**Table 2:** Location of runs of homozygosity island on the genome and list of candidate genes in runs of homozygosity island

Chr	No. SNPs	Physical position (bp)	Length (bp)	Gene symbol	No. Genes
1	5	15,315,574 – 15,406,738	91,164	*SLC2A13*	1
	3	15,772,978 – 15,790,239	17.261	*-*	0
	30	37,177,454 – 37,701,834	524,380	*-*	0
	117	141,255,086 – 144,245,662	2,990,576	*TNFSF13B, ABHD13, LIG4, ARGLU1, EFNB2, SLC10A2, ERCC5, BIVM, POGLUT2, TEX30*	10
2	7	35,537,482 – 35,694,570	157,088	*KCNH8*	1
4	82	40,571,074 – 42,185,328	1,614,254	*-*	0
5	67	2,110,996 – 4,529,658	2,418,662	*NAV2, LEUTX, PRMT3, SLC6A5, NELL1, gga-mir-1775, ANO5, SLC17A6, FANCF, GAS2, SVIP, ANO3, SLC5A12, FIBIN, BBOX1, LGR4, LIN7C, 7SK, BDNF, gga-mir-1760, KIF18A, METTL15P1*	22
	39	41,705,438 – 42,433,307	727,869	*FLRT2*	1
	20	42,495,445 – 42,782,946	287,501	*-*	0
	23	43,363,227 – 43,805,260	442,033	*EML5, TTC8, FOXN3*	3
7	143	6,403,590 – 9,667,318	3,263,728	*HDAC4, NDUFA10, gga-mir-1845, AHR2, COL18A1, SLC19A1, COL6A1, COL6A2, FTCD, YBEY, POFUT2, LSS, S100B, DIP2A, PCNT, C21orf58, KMO, ITGB2, ADARB1, GLS2, STAT1, STAT4, MYO1B, NABP1, CAVIN2, TMEFF2, SLC39A10*	27
8	70	10,320,954 – 12,410,152	2,089,198	*PLA2G4A, PTGS2, PDC, C8H1orf27, TPR, HMCN1, IVNS1ABP, SWT1, TRMT1L, AMY1A, RNPC3, gga-mir-6561, OLFM3, S1PR1, DPH5, SLC30A7, EXTL2, CDC14A, GPR88, RTCA, DBT, LRRC39, TRMT13, SASS6, MFSD14A, SLC35A3*	26
11	47	2,429,665 – 3,897,397	1,467,732	*B3GNT9, TRADD, FBXL8, HSF4, DNAAF1, HSDL1, MBTPS1, SLC38A8, NECAB2, ESRP2, NFATC3, DUS2, DDX28, GALR1L, DPEP2, SLC12A4, SLC6A2, LPCAT2, MMP2, IRX6*	20

Chr, chromosome; No.SNPs, number of single nucleotide polymorphisms; No. genes, number of genes.
